# Supplementary material for: Continuous-wave electrically pumped multi-quantum-well laser based on group-IV semiconductors
Source: Nat Commun. 2024 Dec 3;15:10502. doi: 10.1038/s41467-024-54873-z (PMC11615259; doi:10.1038/s41467-024-54873-z)
Supplement: Supplementary file 1 — Supplementary Information [file 41467_2024_54873_MOESM1_ESM.pdf]

Continuous-wave electrically pumped multi-quantum-well laser based on group-IV semiconductors

Lukas Seidel[1*‡], Teren Liu[2‡], Omar Concepción[2], Bahareh Marzban[3,4], Vivien Kiyek[2,5], Davide Spirito[6,7], Daniel Schwarz[1], Aimen Benkhelifa[1], Jörg Schulze[8], Zoran Ikonic[9], Jean-Michel Hartmann[10], Alexei Chelnokov[10], Jeremy Witzens[3], Giovanni Capellini[6,11], Michael Oehme[1], Detlev Grützmacher[2], Dan Buca[2*]

[1]Institute of Semiconductor Engineering, University of Stuttgart, 70569, Stuttgart, Germany

[2]Peter Gruenberg Institute 9 (PGI-9) and JARA-Fundamentals of Future Information Technologies, Forschungszentrum Juelich, 52428, Juelich, Germany

[3]Institute of Integrated Photonics, RWTH Aachen, 52074, Aachen, Germany

[4]Present address: Institute for Quantum Electronics, ETH Zürich, 8093, Zürich, Switzerland

[5]Present address: Institute of Energy Materials and Devices (IMD-2), Forschungszentrum Juelich, 52428, Juelich, Germany

[6]IHP - Leibniz Institut für innovative Mikroelektronik, 15236 Frankfurt (Oder), Germany

[7]Present address: BCMaterials, Basque Center for Materials, Applications and Nanostructures, UPV/EHU Science Park, 48940 Leioa, Spain

[8]Chair of Electron Devices, Friedrich-Alexander-University, 91058, Erlangen, Germany

[9]Pollard Institute, School of Electronic and Electrical Engineering, University of Leeds, Leeds LS2 9JT, UK

[10]Université Grenoble Alpes, CEA, LETI, 38054 Grenoble, France.

[11]Department of Sciences, Università Roma Tre, 00146, Roma, Italy

‡ These authors contributed equally

*corresponding authors: lukas.seidel@iht.uni-stuttgart.de, d.m.buca@fz-juelich.de

Estimation of CW output power

To estimate the output power, the following setup was used. The sample was glued to a PCB and mounted in a vacuum chamber onto the cold head from a closed-cycle Helium cryostat. Electrical connection was done by bonding from the contact pads to the PCB, which is connected via coaxial cables to a Keithley 2601A source-measure-unit. A direct current was applied for 100 ms. The emitted light is picked up from above the device with a multi-mode fiber having a core diameter of 300μm. The fiber is connected to a NIRQuest 512-2.5 spectrometer with no entrance slit, resulting in a resolution of ~14 nm. Calibration of the setup was done using a HL-3 plus-CAL-EXT calibration lamp with cosine-corrector. The mean spectrum of 2000 measurements with an integration time of 100 ms and the range of integration (25 nm) for the lowest possible temperature of 10 K at a current density of 12 kA/cm^2 is shown in Supplementary Fig. 1.

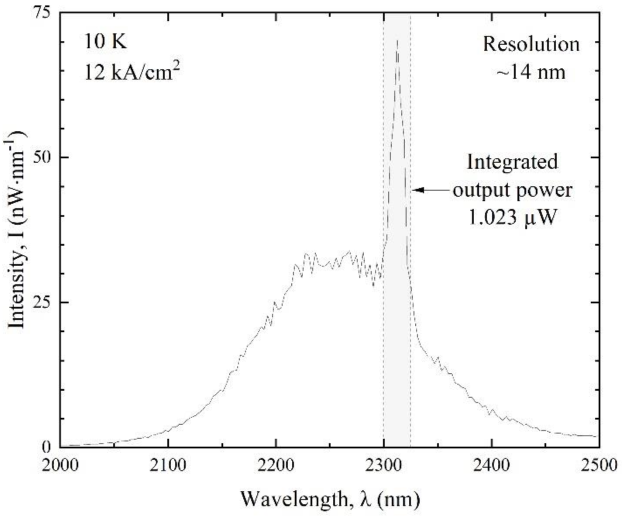

Supplementary Figure 1. Absolute calibrated lasing spectrum: *Spectrum of a micro-disk laser with a radius of 5 μm at a temperature of 10 K and a pumping current density of 12 kA/cm^2. The gray area marks the range for integration.*

CW laser parameters

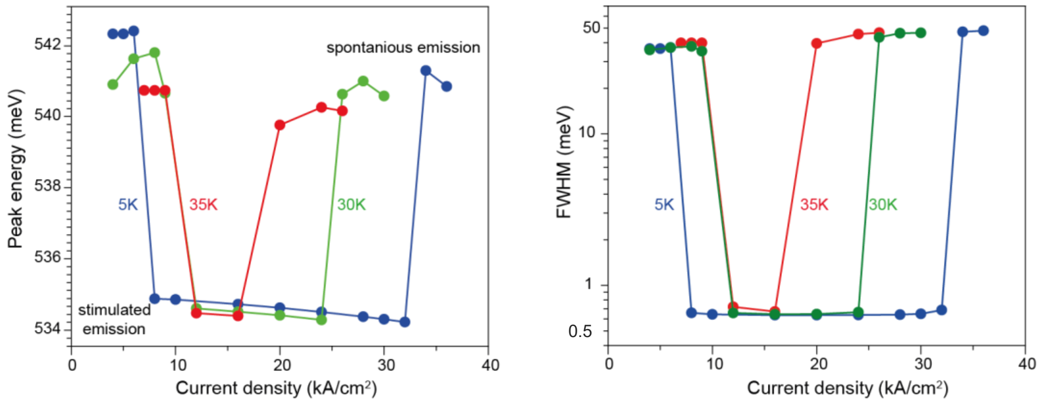

Supplementary Figure 2. Evolution of CW laser parameters: *Peak energy and FWHM of the EL emission as a function of the CW injection current density.*

Temperature dependence of the band-gap

Supplementary Fig. 3a presents a schematic image of a large mesa GeSn/SiGeSn MQW light emitting diode (LED) with a radius of 40 µm. Its temperature-dependent electro-luminescence (EL) spectra for an injection current density of 0.5 kA/cm^2 are plotted in Supplementary Fig. 3c. The typical broadband optical emission is centered around 0.535 eV at T = 5K and red-shifts as the temperature increases, in line with the expected bandgap temperature dependence (Supplementary Fig. 3b). The EL intensity is very strong at 5K and rapidly decreases above 30 K. At 50 K, it is ~40% of that at 5K. At 100K, it is only ~10% of it. This is the consequence of the low band-offset energies for the conduction band (CB) and valence band (VB) at the well-barrier interfaces.

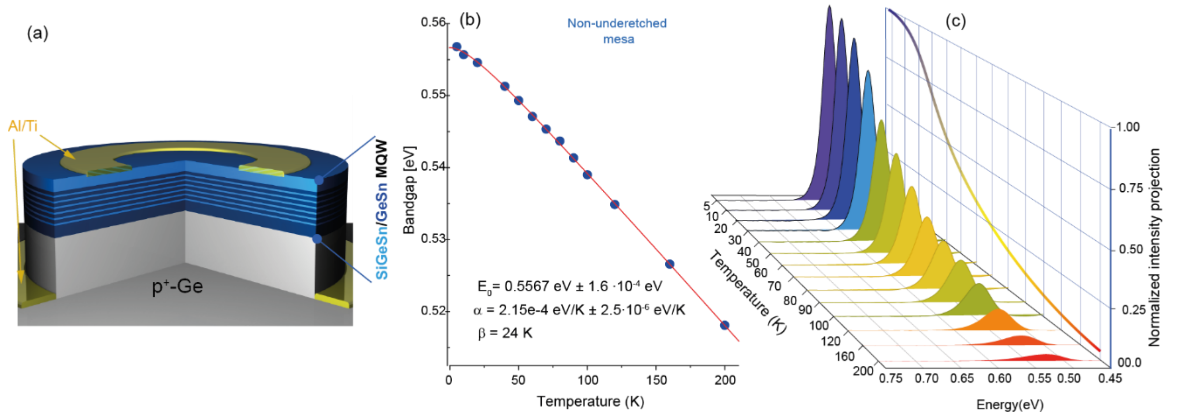

Supplementary Figure 3. Large device LED emission*: (a) Schematic view of the large GeSn/SiGeSn MQW LED. (b) The temperature-dependent bandgap and Varshni fit. (c) Temperature-dependent EL of the LED. The curve to the right summarizes the normalized EL decay vs. temperature.*

For the laser diodes, the partial under-etch of the micro-disk cavity results in some relaxation of the residual compressive strain and thus to some bandgap reduction. Two temperature-dependent sets of EL spectra are taken: at a low injection current <0.4 kA/cm^2 the EL is dominated by emission from the under-etched region, which has the lowest bandgap. At medium injection current levels, the central part of the disk dominates the EL emission.

Both temperature-dependent data sets, with measurements up to 70 K, are used to fit the Varshni coefficients.

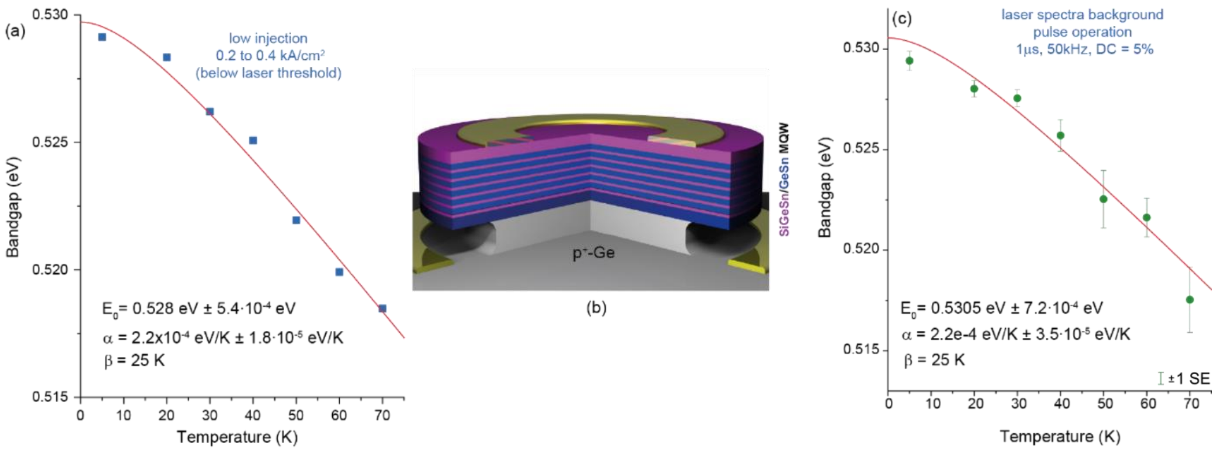

Supplementary Figure 4. Spontaneous LED emission of laser diodes*: The temperature-dependent bandgap and Varshni fit. (a) for low injection currents and (c) background emission of the laser spectra under large current injection. (b) image of the under-etched GeSn/SiGeSn microdisk laser diode.*

Pulsed laser data at 5K

Supplementary Fig. 5a shows a set of ten laser spectra from a GeSn MQW microdisk laser with a radius of 5 μm, taken at 5 K for a current injection ranging from CW down to pulse widths of 165 ns under a constant peak injection current of 20 mA (25 kA/cm^2). Lasing occurs under all these pumping conditions. As an example, the evolution from spontaneous to stimulated emission is shown for 1 ms current pulses at a 500 Hz repetition rate (Duty Cycle 50%) in Supplementary Fig. 5b. The L-I characteristics for three pump pulse lengths of 100 ns, 10 μs, and 1 ms are plotted as functions of the injection current in Supplementary Fig. 5c. Emission intensities are scaled in pulsed mode according to the duty cycle (DC) to enable comparisons between datasets: 0.5% for 100 ns pulses and 50% for 10 μs and 1 ms pulses. The 50% DCs are the maximum possible for the electronic driver setup used for pulsed experiments. The S-shape of the L-I characteristics, specific to the laser emission, is clearly visible. The injection current density at threshold, J_{th}, determined by using the second derivative method (Supplementary Fig. 5d), is about 5 kA/cm^2 (~ 4 mA) for pulses ranging from 100 ns to a few microseconds. It slightly increases to about 5.8 kA/cm^2 (4.7 mA) for millisecond pulses (Supplementary Fig. 5e).

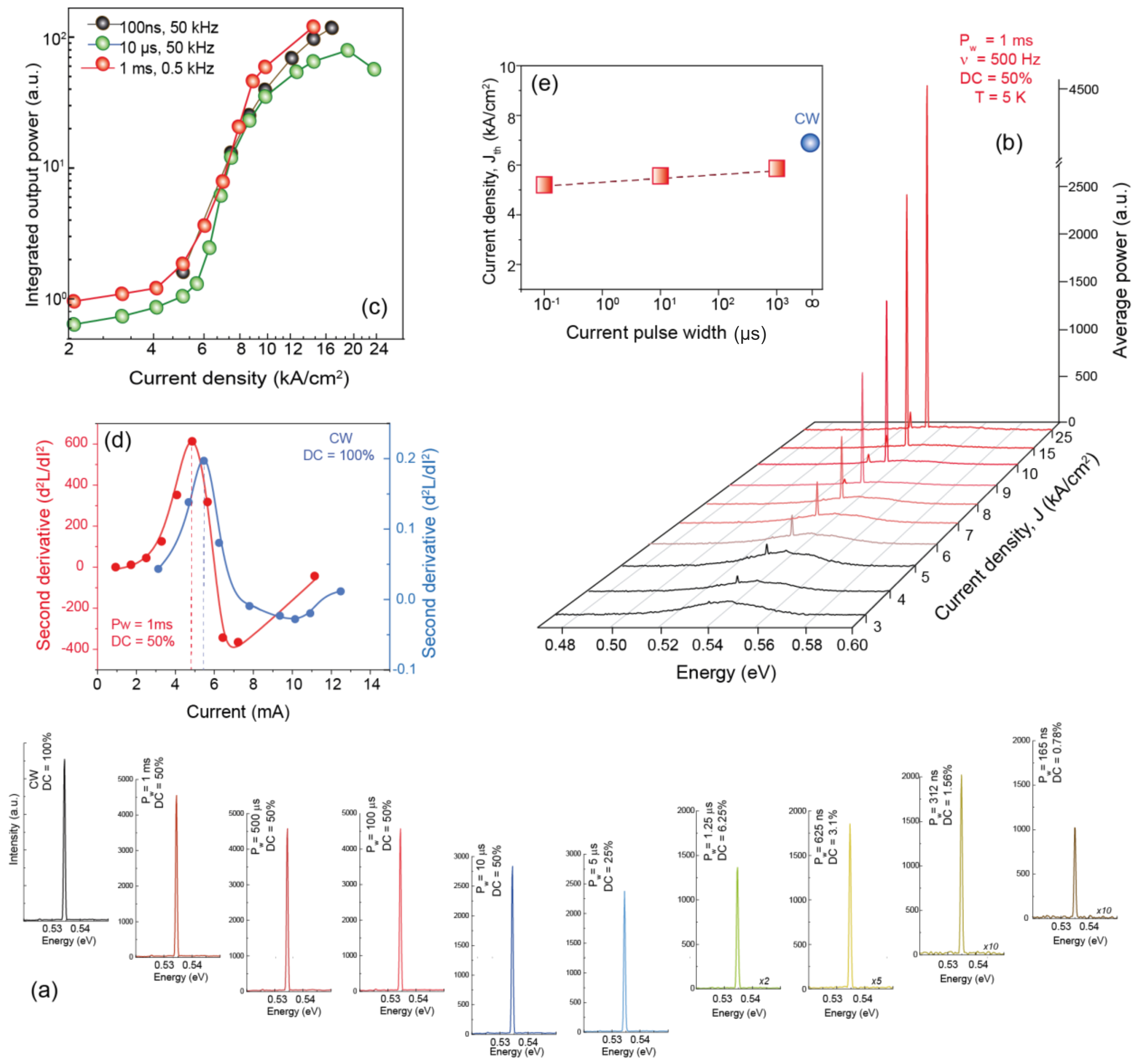

Supplementary Figure 5. Electrically pumped pulsed laser emission at 5K. *(a) A set of 10 laser spectra taken at 5K over a broad range of current injection conditions from CW down to 165 ns pulses. (b) Series of spectra showing the transition from spontaneous to laser emission for 1 ms pulses and a DC of 50%. (c) S-curves on a log-log scale at 5 K for different pumping conditions. (d) Second derivative of the L-I characteristics used for laser threshold extraction for CW and 1 ms pulse pumping conditions. (e) Laser threshold dependence on pulse width in the ns to ms range.*

Pulse dynamics

In the lasing regime where heating is not a limiting factor the laser emission intensity increases linearly (slope m = 1) with the pump pulse width, meaning that the laser emission duration equals the pumping time duration.

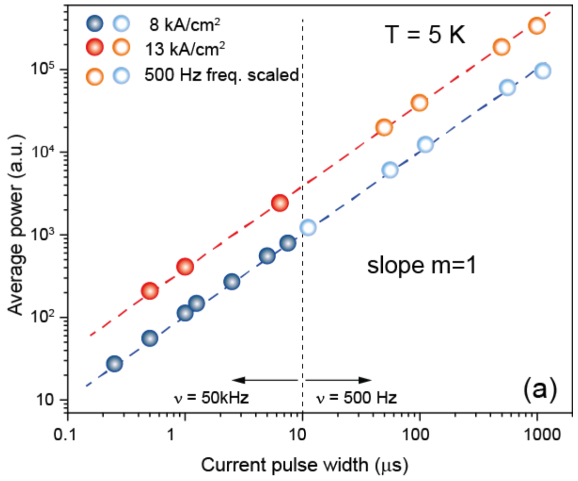

Supplementary Figure 6. Linearity test: *Optical emission at 5K versus the current pulse width at constant frequency for two injection currents. Data points at 500 Hz are frequency scaled.*

The non-linearity versus pulse width at constant frequency indicates that the duration of the stimulated emission is shorter than the pump pulse length, and in the remaining time the optical emission is mostly spontaneous recombination. This can be observed as a broad background in the optical spectra (Fig. 3 in the main article). Generally, disk lasers have a spontaneous emission background arising from the middle part of the disk. The lasing area in disk cavities is small compared with the p-n junctions' area, which is here the total disk area. The spontaneous emission intensity increases with the density of carriers involved in the laser emission, dependent on the injection current and also on the lattice temperature.

The interplay between the pulse length and the frequency allows to investigate the laser action under different conditions. In the main text the spectra evolution at 40K at constant duty cycle is presented (Fig. 3d in the main article). Here we look at the optical emission at 60K function of injection pulse length at constant frequency. At this temperature and under extreme conditions like large current injection (20 mA) and high repetition rate of 50 kHz, no laser emission is measured for pulse above 5 μs. (see Supplementary Fig. 7). However, by reducing the injection pulse duration, meaning reducing the average power as well as increasing the device cooling time between subsequent injection pulses, laser emission is re-established. Interestingly, the spectrum at 5 μs looks very similar to that of the emission before threshold, albeit the injection current density is well beyond the threshold. The spectrum represents a superposition of a laser signal with a large spontaneous emission background, as discussed in the main text for the case of stimulated emission shorter than the pulse length. A further decrease of the pulse length leads to reduction of the background and finally to almost complete extinction of it for 150 ns pulses.

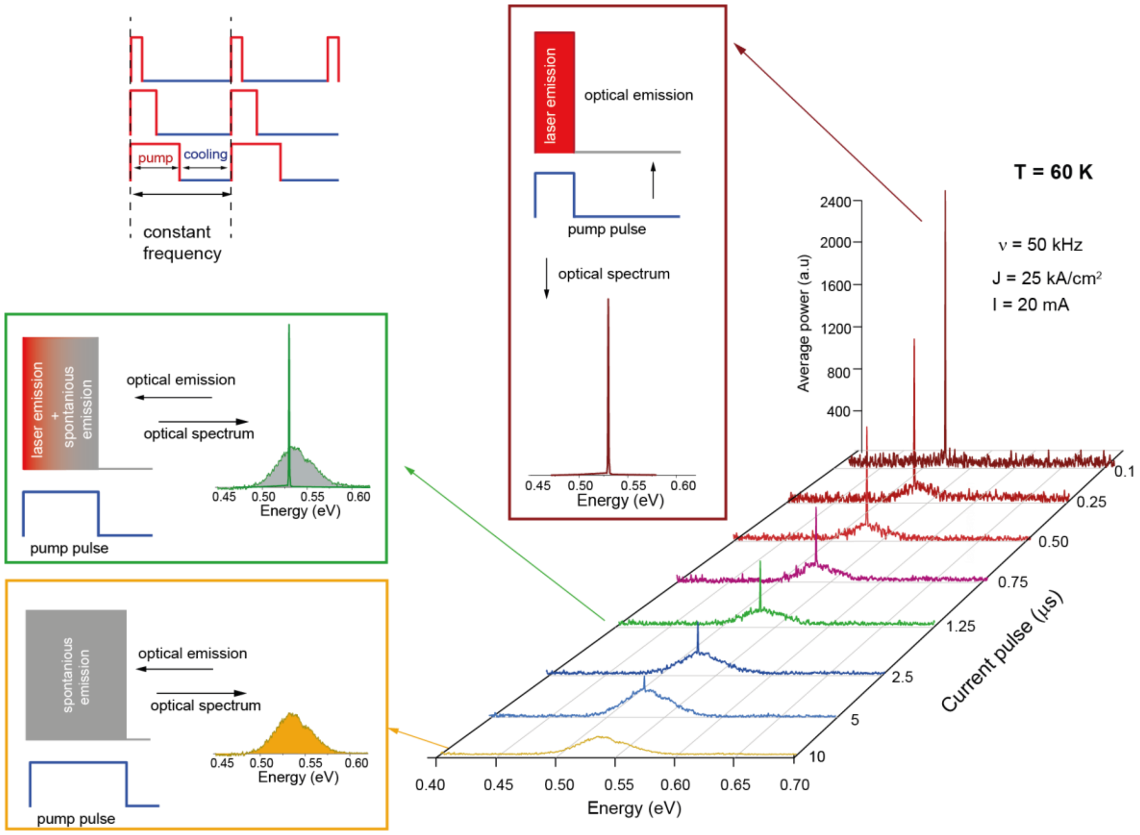

Supplementary Figure 7. Laser emission dynamics: *Optical emission at 60K for different current pulse lengths at constant frequency. The insets schematically indicate the relation between pulse width, laser emission and optical spectrum for 3 different cases: no lasing, partial lasing, and full pulse lasing.*
